# Recruiting neural field theory for data augmentation in a motor imagery brain–computer interface

**DOI:** 10.3389/frobt.2024.1362735

**Published:** 2024-04-17

**Authors:** Daniel Polyakov, Peter A. Robinson, Eli J. Muller, Oren Shriki

**Affiliations:** ^1^ Department of Cognitive and Brain Sciences, Ben-Gurion University of the Negev, Be’er Sheva, Israel; ^2^ Agricultural, Biological, Cognitive Robotics Initiative, Ben-Gurion University of the Negev, Be’er Sheva, Israel; ^3^ School of Physics, The University of Sydney, Sydney, NSW, Australia; ^4^ Brain and Mind Centre, The University of Sydney, Sydney, NSW, Australia

**Keywords:** brain-computer interface (BCI), EEG, motor imagery, data augmentation, neural field theory, common spatial pattern (CSP)

## Abstract

We introduce a novel approach to training data augmentation in brain–computer interfaces (BCIs) using neural field theory (NFT) applied to EEG data from motor imagery tasks. BCIs often suffer from limited accuracy due to a limited amount of training data. To address this, we leveraged a corticothalamic NFT model to generate artificial EEG time series as supplemental training data. We employed the BCI competition IV ‘2a’ dataset to evaluate this augmentation technique. For each individual, we fitted the model to common spatial patterns of each motor imagery class, jittered the fitted parameters, and generated time series for data augmentation. Our method led to significant accuracy improvements of over 2% in classifying the “total power” feature, but not in the case of the “Higuchi fractal dimension” feature. This suggests that the fit NFT model may more favorably represent one feature than the other. These findings pave the way for further exploration of NFT-based data augmentation, highlighting the benefits of biophysically accurate artificial data.

## 1 Introduction

Brain–computer interfaces (BCIs) allow computer and robotic applications to be controlled directly by thoughts (Värbu et al., 2022; Ma et al., 2023). Their widespread use has significantly contributed to aiding individuals with mobility difficulties, artificial limb users, and those affected by paralysis in regaining their motor functions (Mane et al., 2020). BCIs hold particular significance for individuals unable to utilize traditional communication methods, who find themselves entirely locked in due to conditions such as amyotrophic lateral sclerosis, stroke, or traumatic brain injury (Artzi and Shriki, 2018; Nguyen et al., 2018; Willett et al., 2021). Notably, BCI applications extend beyond healthcare, with developments seen in the gaming and defense industries, as well as in the realm of neuro-wellness, catering to cognitive or physical enhancement (Chen et al., 2016).

A typical BCI system consists of several components: a brain-signal acquisition device, such as an electroencephalography (EEG) headset (Värbu et al., 2022), a software module that processes the signals, extracting features and classifying them based on different motor intentions or semantic meanings, and an output device like a monitor, robotic arm, or drone. To produce relevant and distinctive brain signals, users follow mission-specific paradigms, such as motor imagery (MI) (Rahman and Joadder, 2017; Zhang et al., 2021; Hurst and Boe, 2022) or steady-state visual evoked potentials (SSVEPs) (Zhu et al., 2010; Liu et al., 2022). During the training phase, EEG epochs are gathered, aligning with various conditions of a given paradigm’s trials. From these collected epochs, features are extracted and subsequently classified based on their respective conditions. In the operational use of a BCI system, EEG epochs undergo similar stages but are translated into device control commands.

**FIGURE 1:**
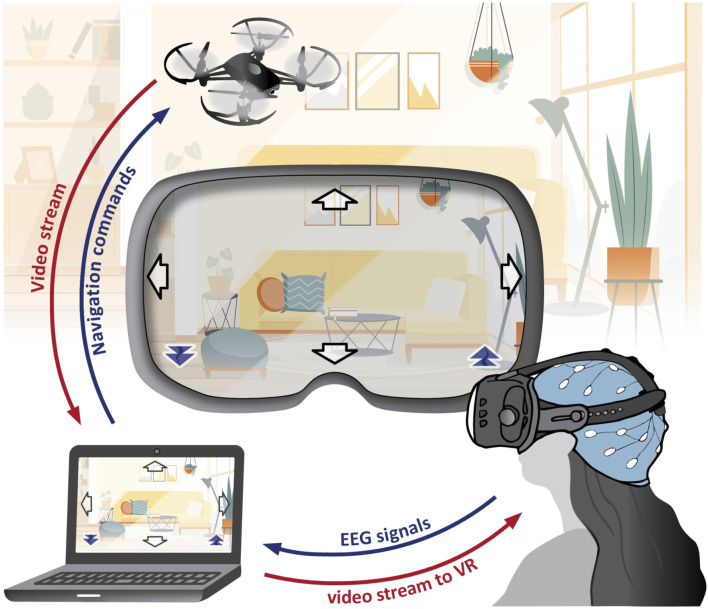
SES-BCI: The setup includes a *DJI-Tello* drone, *Wearable Sensing DSI-24* EEG headset, personal computer, and an optional virtual reality headset. This system simultaneously executes MI and SSVEP paradigms. EEG signals undergo processing to generate navigation commands for the drone. The drone’s video feed presented to the user includes embedded flickering arrows in the corners, acting as stimuli for SSVEPs. In this setup, the SSVEP paradigm is responsible for *forward* and *backward* navigation commands, while the MI paradigm is associated with *right*, *left*, *up*, and *down* navigation commands. (This figure includes a hospital background image by Pikisuperstar from Freepik).

Along with our research, we developed a BCI system for controlling a camera-equipped drone using EEG signals (see Figure 1). It employs both MI and SSVEP simultaneously, creating a hybrid BCI. Within the MI paradigm, we capture EEG patterns associated with imagining body movements, while in SSVEP, we detect patterns arising from looking at flickering stimuli. This approach expands the command repertoire for drone navigation to six distinct actions (fly up, down, left, right, forward, backward), whereas a single paradigm typically offers only two to three conditions with reliable classification accuracy.

The system serves as a surrounding explorer (termed here as “SES-BCI”) for individuals with limited mobility. In a typical scenario, the user comfortably remains in his room, piloting the drone both inside and outside of the house, while simultaneously viewing a live video stream. This setup enables real-time awareness of events, like identifying visitors at the door. For individuals who have completely lost their motor abilities, such a system stands as the sole viable option. This system was not only an integration platform for our developed techniques but also a constant source of inspiration throughout our study.

**FIGURE 2:**
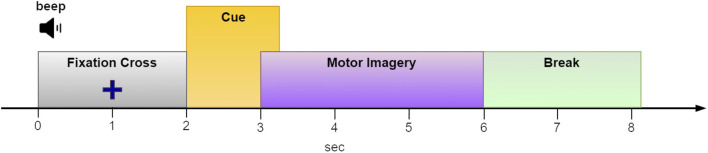
The paradigm sequence utilized in the “2a” dataset from BCI competition IV (Tangermann et al., 2012). We used an EEG segment from *t = 2.5* to *t = 5* s of each epoch for MI classification.

Collecting data for BCI training presents significant challenges. To achieve reliable classification results, a substantial amount of diverse and representative brain signal data is essential. For example, within the MI paradigm, users are often tasked with remaining still and repeatedly imagining different limb movements at least 50 times per limb. Considering an 8-s duration for each MI trial (see Figure 2 for an example) and incorporating four different limbs, the cumulative time required nears 27 min. This process can be strenuous, particularly for individuals with health concerns. However, reducing the number of trials to shorten training sessions might compromise classification accuracy.

Moreover, long-term BCI utilization necessitates daily calibration sessions due to the non-stationarity of brain signals. MI-related brain activity can undergo shifts due to improvements in MI proficiency, or general factors like fatigue or pain. Consequently, portions of the initial training session need to be repeated at the onset of each day when employing the BCI system. This iterative calibration is vital to sustain optimal performance and adapt to the brain’s fluctuating signals (Nicolas-Alonso et al., 2015; Huang et al., 2021).

Training data augmentation (DA) emerges as a potential solution to address these challenges (He et al., 2021; Rommel et al., 2022). The fundamental concept involves conducting a short training session and subsequently adding artificial EEG epochs with similar basic characteristics and some variations. By introducing these augmented epochs, the characteristics of the epochs extend across a broader spectrum of potential values. Consequently, this augmentation elevates the diversity within the training data, thereby contributing to heightened classification accuracy.

Previous research has explored diverse methods of augmenting MI EEG time series. Gubert et al. (2020) expanded the common spectral spatial patterns approach to augment data within a two-condition MI task, resulting in a notable 5% increase in average classification accuracy (Gubert et al., 2020). Lee et al. (2021) employed ensemble empirical mode decomposition to augment a 2-condition MI training set, achieving an impressive over 8% enhancement in classification accuracy (Lee et al., 2021). Conversely, Zhang et al. (2018) introduced Gaussian noise into the EEG signal within the frequency domain to augment a 4-condition MI training set, leading to a more modest improvement of just 2.3% (Zhang et al., 2018). Additionally, the rise of deep learning in the past decade has provided valuable tools for DA. Methods integrating generative adversarial networks and autoencoders demonstrated accuracy improvements exceeding 10% (Zhang et al., 2020; Fahimi et al., 2021). However, notably, none of these approaches have utilized a physiological model to generate EEG data–a direction that remains unexplored in the realm of EEG DA methodologies.

**FIGURE 3:**
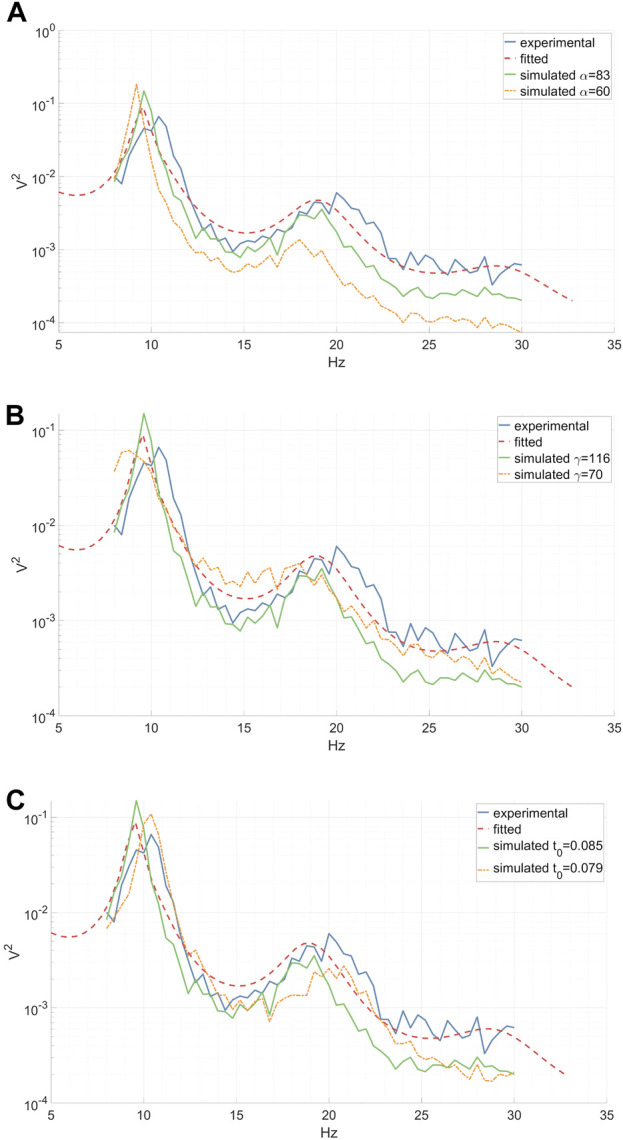
Experimental (inter-epoch average), fitted and simulated power spectra of a right-hand MI CSP source signal between 8 and 30 Hz. Simulated spectra are presented for two distinct parameter values. **(A)** A reduction in the synaptic decay-time constant *α* diminishes the total spectral power, especially affecting the high frequencies. **(B)** A decrease in the cortical damping *γ* results in a decline in the EEG resonant frequency alpha and its peak shift towards the lower frequencies, along with a slight flattening of the beta peak. **(C)** A decrease in the corticothalamic propagation delay *t*_*0*_ leads to a shift of the EEG resonant frequencies alpha and beta peaks towards higher frequencies. **(A–C)**.

The utilization of a physiologically-inspired model for EEG DA offers several distinct advantages. Firstly, it guarantees that the output signals will look like realistic EEG signals. Secondly, it allows for control over the output by manipulating model parameters, enabling the alteration of the signal. Each parameter corresponds to a physiological attribute, e.g., “peak-frequency-location” parameter, ensuring that modifying a parameter yields a precise effect on the output signal (see Figure 3C). Moreover, employing a physiological model ensures that any modified signal remains within the bounds of physiological ranges. When a model mirrors physiological constraints, the distributions of the characteristics within the output signal closely resemble those found in actual physiological data. For example, the distribution of peak frequency locations is non uniform, and a linear change in their control parameter causes an exponential shift in their location.

**FIGURE 4:**
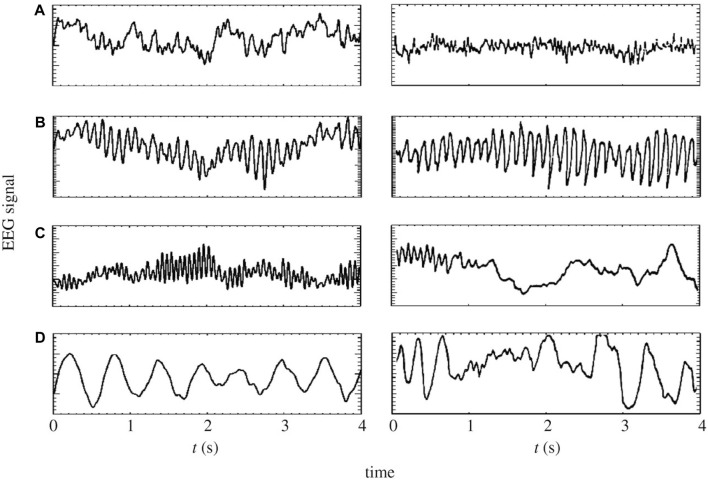
On the left panels: model generated time-series of **(A)** eyes-open resting state, **(B)** eyes-closed resting state, **(C)** sleep-stage 2 and **(D)** sleep-stage 4. On the right panels: corresponding time series from human subjects (Penfield and Jasper, 1954; Nunez, 1995; Robinson et al., 2005).

In this study, we employ a physiological model based on a corticothalamic system, grounded in the neural field theory (NFT) (Robinson et al., 2005; Deco et al., 2008), to augment MI EEG data. NFT is a robust framework widely used to model diverse brain activities, spanning from sleep stages (Rowe et al., 2004; Fulcher et al., 2008; Abeysuriya et al., 2015) (see Figure 4), through epileptic seizures (Breakspear et al., 2006; Nevado-Holgado et al., 2012), and up to abnormalities induced by tumors (O’Connor and Robinson, 2005). Moreover, it has the capacity to capture and replicate these phenomena through EEG data (O’Connor and Robinson, 2004). In the realm of BCI, NFT has been applied to model event-related potentials (Rennie et al., 2002; Kerr et al., 2008; Mukta et al., 2019) and SSVEPs (Roberts and Robinson, 2012; Alinejad et al., 2020), but its application in motor imagery has remained unexplored until now.

To generate artificial EEG signals for MI training data augmentation, we fitted the corticothalamic NFT model (CTM) (Robinson et al., 2002; Robinson et al., 2005; Kerr et al., 2008; Abeysuriya et al., 2015) to MI EEG data obtained from a short training session. We introduced variability in the generated signals by jittering the model parameters. To assess the efficacy of our DA method, we conducted evaluations on the widely used ‘2a’ dataset from BCI Competition IV (Tangermann et al., 2012). The results reveal an increase in accuracy across several scenarios following the implementation of our proposed DA method, emphasizing its viability and promising potential.

## 2 Materials and methods

### 2.1 Neural field theory

Neural-field modeling (Robinson et al., 2001b; Robinson et al., 2004; Robinson et al., 2005) stands as a tool for constructing physiologically-inspired brain models capable of predicting various multiscale measures of brain activity. This approach captures a continuum of corticothalamic activity by simulating the local dynamics in each population and employing wave equations to describe the propagation between these populations (O’Connor and Robinson, 2004). The model’s parameters encompass various biophysically meaningful quantities, such as synaptic strengths, excitatory and inhibitory gains, propagation delays, synaptic and dendritic time constants, and axonal ranges. NFT represents a bottom-up approach to whole-brain modeling, which involves averaging over microstructure to derive mean-field equations.

The corticothalamic NFT model, as introduced by Robinson et al. (2002) and widely adopted thereafter (Robinson et al., 2002; Robinson et al., 2005; Kerr et al., 2008; Abeysuriya et al., 2015), effectively simulates spatiotemporal EEG signals and their spectra. Moreover, in the common case of spatially uniform steady-state activity, it allows for the analytical computation of the power spectrum of the model (Robinson et al., 2005; van Albada et al., 2010). Therefore, NFT provides a practical way for fitting EEG spectra and simulating time series data (Abeysuriya et al., 2015; Sanz-Leon et al., 2018). In this study, we employ the CTM in its original form, leveraging these inherent capabilities.

**FIGURE 5:**
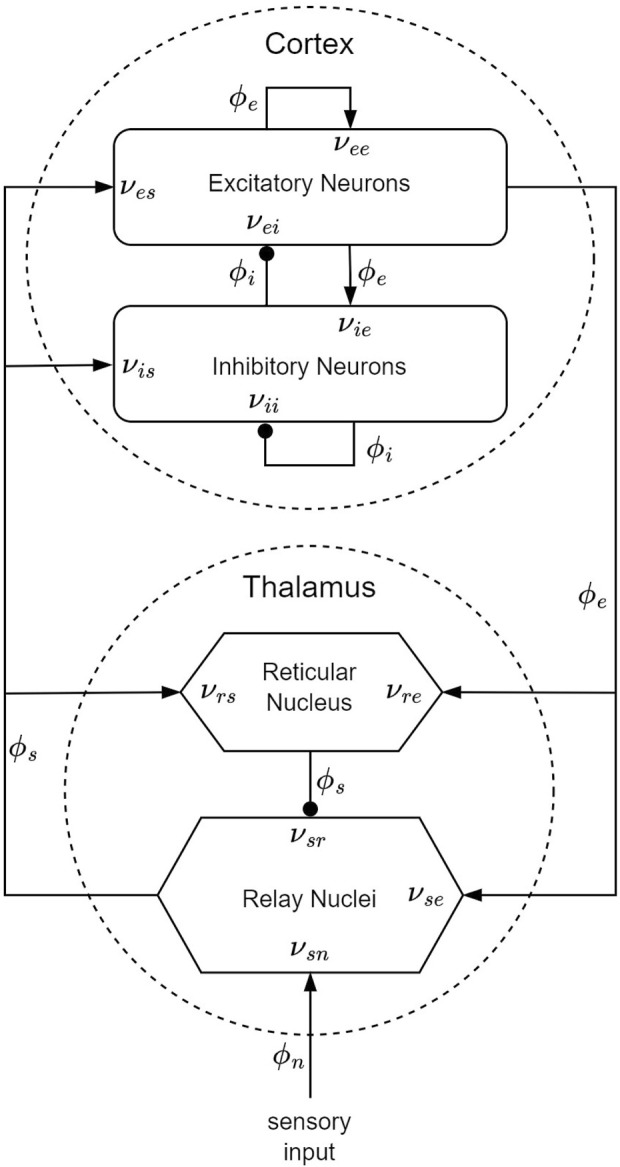
CTM diagram: the neural populations shown are cortical excitatory, *e*, and inhibitory, *i*, thalamic reticular nucleus, *r*, and thalamic relay nuclei, *s*. The parameter *ν*_*ab*_ quantifies the strength of the connection from population *b* to population *a*. Excitatory connections are indicated by pointed arrowheads, while inhibitory connections are denoted by round arrowheads.

Within the framework of the CTM, four distinct neural populations are involved, with key connectivities, illustrated schematically in Figure 5. These populations comprise excitatory (*e*) and inhibitory (*i*) cortical neurons, thalamic relay nuclei neurons (*s*), thalamic reticular nucleus neurons (*r*), and sensory inputs (*n*). Each of these populations has a soma potential *V*_*a*_ (**r**, *t*) [V] that is influenced by contributions *ϕ*_*b*_ from presynaptic populations, and generates outgoing neural activity *ϕ*_*a*_ (**r**, *t*). (Here and further, the sub-indexes *a* and *b* refer to generic populations, which, for example, could be *r* and *e*.) The neural field *ϕ*(**r**, *t*) [s^−1^] represents a spatiotemporal neural activity propagating among populations when averaged across scales of approximately 0.1 mm.

The dendritic spatiotemporal potential *V*_*ab*_ [V] is linked to the input *ϕ*_*b*_ through Eq. 1. The parameter *ν*_*ab*_ = *s*_*ab*_ *N*_*ab*_ [V ⋅s] represents the strength of the connection from populations *b* to *a*, where *N*_*ab*_ is the mean number of synapses per neuron *a* from neurons of type *b*, and *s*_*ab*_ [V ⋅s] is the mean time-integrated strength of soma response per incoming spike. The parameter *τ*_*ab*_ [s] refers to the one-way corticothalamic time delay, and *D*_*a*_(*t*) is a differential operator, as described in Eq. 2. Here, 1/*α* and 1/*β* denote the characteristic decay time and rise time, respectively, of the soma response within the corticothalamic system.

$$D_{a}\left(t\right)V_{ab}\left(r,t\right)=\nu_{ab}\phi_{b}\left(r,t-\tau_{ab}\right)$$ (1)

$$D_{a}\left(t\right)=\frac{1}{\alpha \beta }\frac{d^{2}}{dt^{2}}+\left(\frac{1}{\alpha }+\frac{1}{\beta }\right)\frac{d}{dt}+1$$ (2)

The soma potential *V*_*a*_ is determined as the sum of its dendrite potentials, as outlined in Eq. 3. This potential undergoes some smoothing effects attributed to synaptodendritic dynamics and soma capacitance. Furthermore, the population generates spikes at a mean firing rate *Q*_*a*_ [s^−1^], which is related to the soma potential through a sigmoid function *S*(*V*_*a*_) (relative to the resting state), as shown in Eq. 4. In this equation, *Q*_max_ denotes the maximum firing rate, while *θ* and $\sigma^{\prime }\cdot \pi /\sqrt{3}$ correspond to the mean and the standard deviation, respectively, of the firing threshold voltage.

$$V_{a}\left(r,t\right)=\underset{b}{\sum }V_{ab}\left(r,t\right)$$ (3)

$$Q_{a}=S\left(V_{a}\right)=\frac{Q_{\text{max}}}{1+e^{-\left(V_{a}-\theta \right)/\sigma^{\prime }}}$$ (4)

The field *ϕ*_*a*_ approximately follows a damped wave equation with a source term *Q*_*a*_, as detailed in Eq. 5, delineating its propagation along the axon. The differential operator *D*_*a*_ (**r**, *t*) is defined in Eq. 6, where *v*_*a*_ [m ⋅s^−1^] represents the propagation velocity, *r*_*a*_ [m] denotes the mean range, and *γ*_*a*_ = *v*_*a*_/*r*_*a*_ [s^−1^] signifies the damping rate.

$$D_{a}\left(r,t\right)\phi_{a}\left(r,t\right)=Q_{a}\left(r,t\right)$$ (5)

$$D_{a}\left(r,t\right)=\frac{1}{\gamma_{a}^{2}}\frac{\partial^{2}}{\partial t^{2}}+\frac{2}{\gamma_{a}}\frac{\partial }{\partial t}+1-r_{a}^{2}\nabla^{2}$$ (6)

In this model, only *r*_*e*_ is large enough to induce notable propagation effects. Consequently, the fields of other populations can be approximated as *ϕ*_*a*_ (**r**, *t*) = *S* [*V*_*a*_ (**r**, *t*)]. Additionally, we assume that the only non-zero time delays between populations are *τ*_*es*_, *τ*_*is*_, *τ*_*se*_, and *τ*_*re*_ = *t*_0_/2, where *t*_*0*_ is the total time it takes to traverse the corticothalamic loop. It is important to note that Eq. 5 encompasses the corticocortical time delays, as the wave equation inherently accounts for delays arising from propagation across the cortex. To further simplify the model, we assume random intracortical connectivity, leading to *N*_*ib*_ = *N*_*eb*_ for all *b* (Braitenberg and Schüz, 1998). This assumption implies that the connection strengths are also symmetric, resulting in *ν*_*ee*_ = *ν*_*ie*_, *ν*_*ei*_ = *ν*_*ii*_, and *ν*_*es*_ = *ν*_*is*_ (Robinson et al., 2005; Abeysuriya et al., 2015). Numerical integration (Sanz-Leon et al., 2018) or, when feasible, analytical integration (Robinson et al., 1997; Robinson et al., 2001a) of NFT equations produces a spatiotemporal activity signal that propagates across the cortical surface. For instance, to solve Eq. 1 we can integrate *ϕ*_*b*_ with an impulse response kernel *L*(*t*) (Eq. 8) over time.

$$V_{ab}\left(r,t\right)=\int_{-\infty }^{t}L\left(t-t^{\prime }\right)\nu_{ab}\phi_{ab}\left(r,t^{\prime }-\tau_{ab}\right)\;dt^{\prime }$$ (7)

$$L\left(t\right)=\Theta \left(t\right)\cdot \left\{\begin{array}{cc} \frac{\alpha \beta }{\beta -\alpha }\left(e^{-\alpha t}-e^{-\beta t}\right)\; & ,\alpha \neq \beta \\ \alpha^{2}te^{-\alpha t}\; & ,\alpha =\beta \end{array}\right.$$ (8)

$$\Theta \left(t\right)\;\;is\;the\;Heaviside\;step\;function$$

### 2.2 CTM EEG power spectrum

In scenarios of spatially uniform steady-state activity, it is possible to analytically compute the power spectrum of the model, eliminating the necessity for numerical integration. The steady state is attained by setting all time and space derivatives to zero. Employing the first term of the Taylor expansion enables a linear approximation of all potential perturbations from the steady state. Applying a Fourier transform to the model equations under these conditions yields Eq. 9 for the dendritic component and Eq. 10 for the axonal component. Within these equations, *ω* = 2*πf* denotes the angular frequency, *k* = 2*π*/*λ* signifies the wave vector (*λ* is the wavelength), and $V_{a}^{\;(0)}$ represents the steady-state potential (Abeysuriya et al., 2015).

Dendritic equations in the frequency domain:

$$V_{ab}\left(k,\omega \right)=\nu_{ab}\phi_{b}\left(k,\omega \right)L\left(\omega \right)e^{i\omega \tau_{ab}}$$ (9)

$$L\left(\omega \right)={\left(1-\frac{i\omega }{\alpha }\right)}^{-1}{\left(1-\frac{i\omega }{\beta }\right)}^{-1}$$

Axonal equations in the frequency domain:

$$D_{a}\left(k,\omega \right)\cdot \phi_{a}\left(k,\omega \right)=\rho_{a}V_{a}\left(k,\omega \right)$$ (10)

$$\begin{array}{ll} D_{a}\left(k,\omega \right) & =k^{2}r_{a}^{\;2}+{\left(1-\frac{i\omega }{\gamma_{a}}\right)}^{2}\\ \rho_{a} & =\frac{dS\left(V_{a}^{\left(0\right)}\right)}{dV_{a}} \end{array}$$

Using Eq. 3, we can write Eq. 10 as:

$$D_{a}\left(k,\omega \right)\cdot \phi_{a}\left(k,\omega \right)=\rho_{a}\underset{b}{\sum }V_{ab}\left(k,\omega \right)=\underset{b}{\sum }J_{ab}\phi_{b}\left(k,\omega \right)$$ (11)

$$J_{ab}=\rho_{a}\nu_{ab}L\left(\omega \right)e^{i\omega \tau ab}=G_{ab}L\left(\omega \right)e^{i\omega \tau_{ab}}$$

Then we can represent the interactions among the different populations within the CTM in matrix form:

$$\begin{array}{ll} \left[\begin{array}{cccc} D_{e} & 0 & 0 & 0\\ 0 & D_{i} & 0 & 0\\ 0 & 0 & D_{r} & 0\\ 0 & 0 & 0 & D_{s} \end{array}\right]\cdot \left[\begin{array}{c} \phi_{e}\\ \phi_{i}\\ \phi_{r}\\ \phi_{s} \end{array}\right] & =\left[\begin{array}{cccc} J_{ee} & J_{ei} & 0 & J_{es}\\ J_{ie} & J_{ii} & 0 & J_{is}\\ J_{re} & 0 & 0 & J_{rs}\\ J_{se} & 0 & J_{sr} & 0 \end{array}\right]\\ & \;\cdot \left[\begin{array}{c} \phi_{e}\\ \phi_{i}\\ \phi_{r}\\ \phi_{s} \end{array}\right]+\left[\begin{array}{c} 0\\ 0\\ 0\\ J_{sn}\phi_{n} \end{array}\right] \end{array}$$ (12)

Eq. 12 can also be written in a compact form, when **J** ^***⋆***^ ***ϕ*** ^**⋆**^ is the external input to the CTM:

$$D\phi =J\phi +J^{⋆}\phi^{⋆}$$ (13)

By solving Eq. 12, considering all the previously mentioned assumptions regarding *D*_*a*_, *ν*_*ab*_, and *τ*_*ab*_, we can derive Eq. 14. In this context, the quantities *G*_*ese*_ = *G*_*es*_ *G*_*se*_, *G*_*esre*_ = *G*_*es*_ *G*_*sr*_ *G*_*re*_, and *G*_*srs*_ = *G*_*sr*_ *G*_*rs*_ correspond to the overall gains for the excitatory corticothalamic, inhibitory corticothalamic, and intrathalamic loops, respectively. The firing rate of sensory inputs to the thalamus, *ϕ*_*n*_, is approximated by white noise. Without loss of generality, *ϕ*_*n*_(*ω*) can be set to 1, while only *G*_*sn*_ is subject to variation.

$$\phi_{e}\left(k,\omega \right)=\frac{G_{es}G_{sn}L^{2}e^{\frac{i\omega t_{0}}{2}}}{\left(1-G_{\mathrm{srs}}L^{2}\right)\left(1-G_{ei}L\right)\left(k^{2}r_{e}^{2}+q^{2}r_{e}^{2}\right)}\phi_{n}\left(k,\omega \right)$$ (14)

$$q^{2}r_{e}^{2}={\left(1-\frac{i\omega }{\gamma_{e}}\right)}^{2}-\frac{1}{1-G_{ei}L}\left\{LG_{ee}+\frac{\left[L^{2}G_{\mathrm{ese}}+L^{3}G_{\mathrm{esre}}\right]e^{i\omega t_{0}}}{1-L^{2}G_{\mathrm{srs}}}\right\}$$

The excitatory field *ϕ*_*e*_ is considered a good approximation of scalp EEG signals (Abeysuriya et al., 2015). The EEG power spectrum *P*(*ω*) (Eq. 15) is calculated by integration of |*ϕ*_*e*_ (**k**, *ω*)|^2^ over **k** when the cortex is approximated as a rectangular sheet of size *L*_*x*_ × *L*_*y*_. When considering periodic boundary conditions, this integral transitions into a summation over spatial modes with a discrete *k*. The filter function *F*(*k*) serves as an approximation of the low-pass spatial filtering that occurs due to volume conduction through the cerebrospinal fluid, skull, and scalp.

$$P\left(\omega \right)=\overset{\infty }{\underset{m=-\infty }{\sum }}\overset{\infty }{\underset{n=-\infty }{\sum }}|\phi_{e}\left(k_{x},k_{y},\omega \right){|}^{2}F\left(k\right)\Delta k_{x}\Delta k_{y}$$ (15)

$$\begin{array}{ll} k_{x} & =\frac{2\pi m}{L_{x}}\text{, }k_{y}=\frac{2\pi n}{L_{y}}\text{, }k=\sqrt{k_{x}^{2}+k_{y}^{2}}\\ F\left(k\right) & =e^{-k^{2}/k_{0}^{2}} \end{array}$$

### 2.3 MI EEG signal processing and feature extraction

**FIGURE 6:**
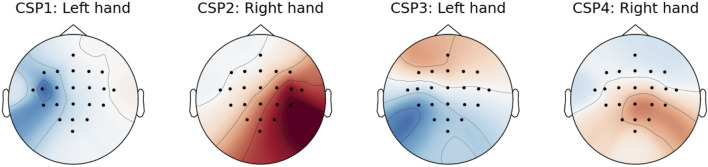
Power topoplots of CSPs fitted to MI EEG epochs. The fitting process aims to enhance the differentiation between right-hand and left-hand MI conditions.

During the MI paradigm, subjects engage in imagery of specific limb movements, inducing activity modulation within their motor cortex (Rahman and Joadder, 2017; Hurst and Boe, 2022). One method to detect MI-related activity within EEG signals is through the application of common spatial patterns (CSPs). This supervised technique decomposes EEG signals into distinct sources, where each source exhibits high variability for a particular MI condition and low variability for others (Koles et al., 1990). An example of CSP topoplots fitted to distinguish between right-hand and left-hand MI can be seen in Figure 6. We set the number of CSPs to 2 ⋅⌈1 + *#conditions*/2⌉ following an empirical exploration of classification accuracy, noting that the accuracy exhibited marginal improvements beyond a certain number of CSPs. Moreover, employing a limited number of CSPs helps prevent overfitting and expedites the DA process. Before applying the CSP technique, the EEG data underwent band-pass filtering within the spectral range of 8–30 Hz. This frequency range is known to capture the characteristic spectral patterns associated with MI.

Two features, total power (TP) and Higuchi fractal dimension (HFD) (Higuchi, 1988) were computed from each CSP in every epoch using the *mne-features* toolbox (Schiratti et al., 2018). These features were chosen for two primary reasons. First, they are commonly employed in MI classification and demonstrate effective discrimination within our data. Second, they are computed using distinct methodologies: TP involves summing the signal power and can be derived from the spectrum (see Eq. 16), while HFD identifies patterns within the samples and is derived from the time series, as described below. During the evaluation of DA performance (see next section), each feature is individually employed to understand how augmentation depends on feature characteristics.

The total power of signal *X* can be calculated from the time series *X*(*n*) or its discrete Fourier transform $\hat{X}\left(l\right)$ using the following expression, while *N* and *T* are the lengths of *X*(*n*) and $\hat{X}\left(l\right)$ , respectively:

$$TP=\frac{1}{N}\underset{n}{\sum }X^{2}\left(n\right)=\frac{1}{T}\underset{l}{\sum }{\left|\hat{X}\left(l\right)\right|}^{2}$$ (16)

The Higuchi fractal dimension of *X*(*n*) is calculated through the following steps:

1. Create new time series $X_{m}^{n}$ by dividing the original time series into non-overlapping segments.

$$X_{m}^{n}=\left\{X\left(m\right),X\left(m+n\right),X\left(m+2n\right),\ldots ,X\left(m+pn\right)\right\},\;\;\;\left(m=1\;\ldots n\right)$$ (17)

$$\begin{array}{ll} p & =\left\lfloor \left(N-m\right)/n\right\rfloor \;\;\;\;\;\; \end{array}$$

2. Compute the length *L*_*m*_(*n*) of each $X_{m}^{n}$ .

$$L_{m}\left(n\right)=\frac{N-1}{n^{2}}\cdot \frac{1}{p}\overset{p}{\underset{i=1}{\sum }}\left|X\left(m+in\right)-X\left(m+\left(i-1\right)n\right)\right|$$ (18)

3. Calculate the average length $L\left(n\right)={\left\langle L_{m}\left(n\right)\right\rangle }_{m}$ .
4. Fit a linear model to *log*_2_ [*L*(*n*)] as a function of *log*_2_(*n*). The slope parameter of the linear model is an estimate of the HFD.

Finally, a linear discriminant analysis (LDA) (Tharwat et al., 2017) was applied to these feature values to classify the MI conditions. Linear discriminant analysis is frequently utilized for classifying CSP-based features due to its ability to leverage feature variance for classification purposes. The classification accuracy was determined as an average of the true positive rates for each condition:

$$Ac=\frac{1}{\#conditions}\cdot \underset{i}{\sum }\left(\left.\frac{\#\;trials\;classified\;as\;condition_{i}}{\#trials}\;\right|\;trial\in condition_{i}\right)$$ (19)

### 2.4 Motor imagery data augmentation

#### 2.4.1 NFT-based augmentation

The DA process for MI training takes place at the level of CSP sources instead of directly augmenting the EEG channel time series. This strategy was chosen over augmenting the EEG channel time series directly for two primary reasons. Firstly, strong correlations exist among numerous EEG channels, leading to significant redundancy in augmented data and potentially suppressing the manifestation of less common low-power activity. However, by employing CSP decomposition, similar activities are grouped together, and distinct spatial sources are assigned to distinct activity patterns. Secondly, by augmenting at the CSP level, the process becomes more efficient and practical compared to augmenting individual channels. Augmenting four to six CSPs is notably faster and more manageable than augmenting 22 channels separately.

**FIGURE 7:**
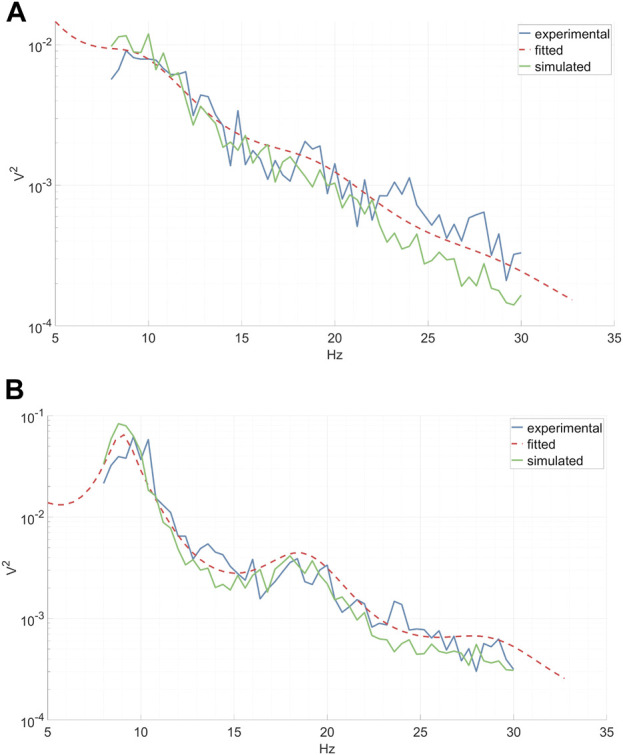
Experimental inter-epoch average EEG power spectrum, analytical power spectrum of a fitted CTM, and a power spectrum calculated from time series simulated by a fitted CTM. Shown are the left-hand MI CSP **(A)** and the corresponding right-hand MI CSP **(B)**.

Each subject’s epochs were grouped according to their respective condition. Subsequently, the power spectra of CSP source signals for each epoch were computed via a fast Fourier transform and then averaged across epochs. The CTM was fitted to each average power spectrum (see the “CTM EEG Power Spectrum” paragraph) utilizing the Markov Chain Monte Carlo algorithm implemented in the *braintrak* toolbox (Abeysuriya and Robinson, 2016). In line with previous studies, we chose to fit the following parameters: connection gains, *G*_*ij*_, corticothalamic delay *t*_*0*_, and the synaptic decay and rise time constants *α*, *β* (Abeysuriya et al., 2015). Artificial time series were then generated from the fitted model, employing the *NFTsim* toolbox (Sanz-Leon et al., 2018). An example of average experimental spectra, fitted analytical spectra, and spectra of the simulated time series associated with left-hand MI CSP and right-hand MI CSP can be seen in Figure 7. The continuous simulated CSP source signals were segmented into epochs, rendering them suitable for subsequent classifier training.

The augmentation process included an optional stage where a few of the fitted parameters, either collectively or separately, underwent jittering before the signal generation phase. This step was implemented to introduce additional variability into the generated data. The aim was to enhance the inherent variability of the generated signals, stemming from the sensory inputs to the thalamic relay nuclei *ϕ*_*n*_ that drive the CTM with white noise. Specifically, jittering was applied to modify the parameters *α* (synaptic decay-time constant [s^−1^]), *γ* (cortical damping [s^−1^]), and *t*_*0*_ (corticothalamic delay [s]). However, other parameters of the fitted model, such as cortical loop gains, were intentionally excluded from the jittering process. This cautious approach was adopted to prevent potential bifurcations within the CTM that could result in a significantly different spectrum.

Jittering of parameters was achieved by adding Gaussian-distributed values *N* (0, 1.5 ⋅ *σ*_*typical*_) to the fitted parameters and drawing new values 10 ⋅ (*augmentation factor*) times during the signal generation stage. The standard deviation *σ*_*typical*_ was set based on previous research findings: *σ*_*typical*_(*α*) = 14, *σ*_*typical*_(*γ*) = 25, *σ*_*typical*_ (*t*_0_) = 0.003 (Rowe et al., 2004). These standard deviation values were increased only by a factor of 1.5 to ensure that they remained within typical parameter ranges and avoided excessive deviation.

#### 2.4.2 Noise-based augmentation

We compared our DA method to a naive augmentation approach that involves noise introduction into feature values. For each MI condition, we calculated the mean *μ*_*feat*_ and the covariance matrix *S*_*feat*_ of the features. Subsequently, new feature values were drawn from a Gaussian distribution *N* (*μ*_*feat*_, 1.5 ⋅ *S*_*feat*_).

### 2.5 Performance evaluation

#### 2.5.1 The “2a” dataset

To benchmark DA performance against a well-known benchmark, we tested it using dataset ‘2a’ from BCI Competition IV. This dataset comprises recordings from nine subjects engaged in MI tasks involving the right hand, left hand, feet, and tongue. The experiment was repeated on two consecutive days. However, given that our research did not focus on BCI stationarity, we chose to treat each day’s data as if it came from a different subject, thereby resulting in a total of 18 distinct subjects for analysis. Each subject provided a total of 288 epochs (72 epochs per condition). However, our evaluation focused solely on the two-condition set, specifically the right/left hand MI tasks. Each epoch had a duration of 6 seconds, with the imagery cue onset at *t = 2* s (*t = 0* marks the beginning of the epoch; see Figure 2). We, therefore, considered the interval between *t = 2.5* and *t = 5* s as the MI period. Epochs that were marked as “bad” were ignored. The EEG data was sampled at 250 Hz and comprised 22 channels positioned on the scalp following the international 10–20 EEG system (Tangermann et al., 2012).

#### 2.5.2 Training, augmentation, and validation sets

**FIGURE 8:**
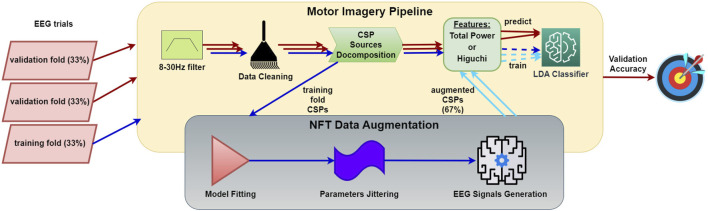
Workflow of MI data augmentation performance evaluation procedure. The process involves creating a small dataset and assessing accuracy using inverse CV, where one fold is reserved for training and the others for testing. The MI pipeline consists of EEG epoch preprocessing, CSP decomposition, feature extraction, and classification. The NFT-based data augmentation process generates artificial CSP time series using a CTM (Robinson et al., 2002; Robinson et al., 2005; Kerr et al., 2008; Abeysuriya et al., 2015) fitted to the CSP time series from the MI pipeline.

To create a small training set, we randomly partitioned the training epochs into three subsets and employed an inverse 3-fold cross-validation (CV); namely, we used 33% for training and 67% for validation. In the typical augmentation scenario, we augmented the training data by a factor of 2, topping up the total training data to 100%. We calculated the accuracy rates of the following sets: the initial 100% of the epochs employing a regular 7-fold CV (86% for training, 14% for validation), the small set using an inverse CV (33% for training, 67% for validation), and the small set combined with the augmented set involving inverse CV (33% + 33% ⋅ [*augmentation factor*] for training, 67% for validation). These rates were compared against each other, verifying any accuracy improvements for statistical significance with a paired Student’s t-test across subjects. The entire process is illustrated in Figure 8.

## 3 Results

Here, we present the outcomes of the proposed DA approach applied to the ‘2a’ dataset. We experimented with various augmentation strategies defined by distinct augmentation factors and jittered parameters. We observed that the majority of the NFT-driven strategies significantly improved TP feature classification accuracy, while the noise-based DA did not exhibit notable enhancements. Notably, the augmented set’s classification accuracy consistently surpassed that of the small set, but failed to reach the level of the full set accuracy. Intriguingly, no enhancement was noted in HFD feature classification across the various DA strategies. Moreover, we noticed that MI proficiency plays a key role in DA success, with subjects who initially demonstrated plausible classification accuracy more likely to benefit from DA enhancements.

While analyzing the results, we excluded subjects whose MI classification accuracy fell below chance level to ensure that the DA was applied to separable data. Additionally, we excluded subjects who did not display a reduction in accuracy with the small set since we wanted to show how DA compensates for the epochs deducted from the full set. In other words, subjects were filtered out according to the following criteria: *full set Ac* ≤ 0.5, *small set Ac* ≤ 0.5, and *small set Ac* > *full set Ac*. Consequently, out of the 18 subjects in the dataset, 14 subjects in TP feature classification and 11 subjects in HFD remained eligible for DA assessment.

Table 1 showcases the classification accuracy results for TP features across various augmentation strategies. The inter-subject average for the full (100%) training set accuracy was 0.75, while the small (33%) training set accuracy (denoted further as “baseline”) achieved 0.71. Notably, the augmentation strategy using NFT with a factor of 2 (67%) demonstrated a significant improvement in accuracy (Δ*Ac* = 0.01, *p* < 0.05). However, the improvements from other strategies were statistically insignificant. Figure 10A depicts original and augmented TP feature values. The augmentation process introduced diversity into feature values without compromising the linear discrimination between left- and right-hand MI trials.

**TABLE 1:** Performance evaluation results for various data augmentation strategies. The table presents validation set accuracy *Ac* and the accuracy improvement Δ*Ac* obtained for TP feature classification.

DA strategy	**Augmented set** *Ac*	**Small set** Δ*Ac* = *Ac* − 0.715	**Full set** Δ*Ac* = *Ac* − 0.751
small (33%) + 33% NFT	0.726	0.011, *p* = 0.06	−0.025, *p* = 0.011
small (33%) + 67% NFT	0.725	0.01, *p* = 0.043	−0.026, *p* = 0.004
small (33%) + 167% NFT	0.723	0.008, *p* = 0.173	−0.028, *p* = 0.004
small (33%) + 33% NFT jitter [*t*_*0*_]	0.721	0.006, *p* = 0.179	−0.03, *p* = 0.002
small (33%) + 67% NFT jitter [*t*_*0*_]	0.72	0.005, *p* = 0.385	−0.031, *p* = 0.001
small (33%) + 167% NFT jitter [*t*_*0*_]	0.717	0.002, *p* = 0.763	−0.034, *p* = 0.0006
small (33%) + 67% noise	0.719	0.004, *p* = 0.711	−0.032, *p* = 0.029

The exclusion of subjects with low MI proficiency is a common practice in BCI training. To investigate its impact on accuracy improvements with other DA strategies, we defined MI proficiency based on the subject’s baseline accuracy. Through a grid search, we identified that setting an MI proficiency threshold of *Ac* ≥ 0.67 resulted in the highest number of successful DA strategies.

**FIGURE 9:**
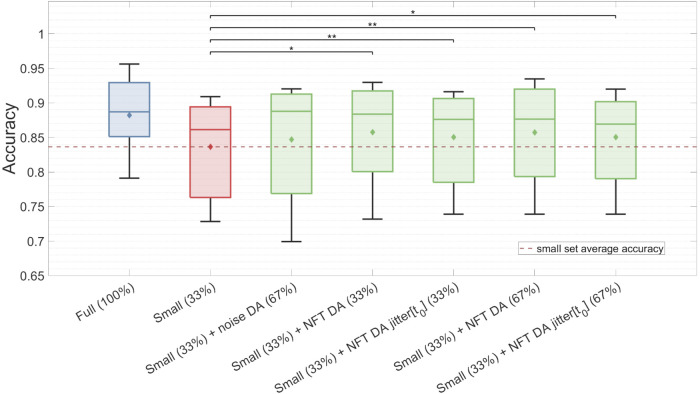
Classification accuracy of the TP feature for the full dataset, the small dataset, and the augmented small dataset using different DA strategies. The classification was conducted on validation sets for subjects with high MI proficiency. Noticeably, NFT-based DA approaches show statistically significant improvements in *Ac*, whereas noise-based DA does not yield significant enhancements.

The findings for the TP feature classification among subjects with high MI proficiency are summarized in Figure 9 and Table 2. NFT-based DA using augmentation factors of 1 (33%), 2 (67%), and 5 (167%) presented significant improvements over the baseline, showcasing the most substantial improvement of Δ*Ac* = 0.022, *p* < 0.05. Notably, smaller augmentation factors led to higher accuracy improvements. Besides, the introduction of parameter jittering appeared to negatively impact augmentation performance, except for jittering of *t*_*0*_, which exhibited an improvement, but was still lower than the improvement observed in DA without any jittering. It is worth mentioning that MI amateurs with *Ac* < 0.67 did not demonstrate significant improvement above the baseline for any of the DA strategies. Although noise-based DA exhibited some improvement, it was not statistically significant. The results in Figure 9 and Table 2 are based on 7 out of 14 subjects, as the others demonstrated a proficiency below 0.67. The high proficiency group’s baseline accuracy was 0.84, while the full set accuracy was 0.88.

**TABLE 2:** Performance evaluation results for various data augmentation strategies, as conducted on subjects with **high MI proficiency**. The table presents validation set accuracy *Ac* and the accuracy improvement Δ*Ac* obtained for TP feature classification.

DA strategy	**Augmented set** *Ac*	**Small set** Δ*Ac* = *Ac* − 0.836	**Full set** Δ*Ac* = *Ac* − 0.882
small (33%) + 33% NFT	0.858	0.022, *p* = 0.013	−0.024, *p* = 0.169
small (33%) + 67% NFT	0.857	0.021, *p* = 0.003	−0.025, *p* = 0.134
small (33%) + 167% NFT	0.853	0.017, *p* = 0.012	−0.029, *p* = 0.092
small (33%) + 33% NFT jitter [*t*_*0*_]	0.85	0.014, *p* = 0.003	−0.032, *p* = 0.06
small (33%) + 67% NFT jitter [*t*_*0*_]	0.85	0.014, *p* = 0.017	−0.032, *p* = 0.07
small (33%) + 167% NFT jitter [*t*_*0*_]	0.848	0.012, *p* = 0.102	−0.034, *p* = 0.041
small (33%) + 67% noise	0.847	0.011, *p* = 0.327	−0.035, *p* = 0.114

**FIGURE 10:**
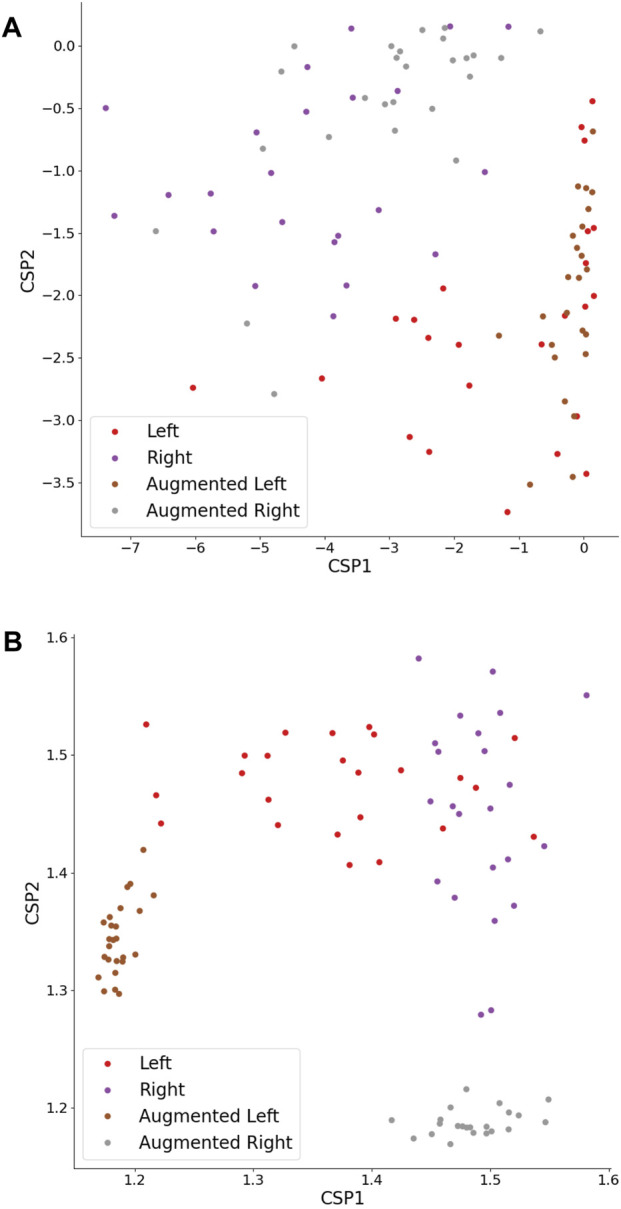
TP **(A)** and HFD **(B)** features extracted from CSPs of original and augmented time series. The CSPs were augmented by a factor of 1, employing an NFT model. The left- and right-hand MI trials maintain linear separability.

As previously mentioned, HFD feature classification did not exhibit sensitivity to any DA strategy, indicating that all augmented set accuracies remained around the baseline, regardless of the *Ac* threshold. It can be seen in Figure 10B that the DA procedure did not introduce diversity to feature values, but instead created features with stereotypical values falling outside the typical feature range. Intriguingly, the HFD feature demonstrated a full set accuracy of 0.79 and a baseline accuracy of 0.75, surpassing that of the TP feature. These observations suggest that the underlying cause lies within the inherent nature of the HFD feature rather than other contributing factors.

### 3.1 Spectral effect of parameter jittering

We investigated the impact of jittering *α*, *γ*, and *t*_*0*_ model parameters on the power spectrum of the simulated output signal, thereby characterizing the diversity within the augmented data. Our analysis revealed that altering the synaptic decay-time constant *α* significantly influenced the total spectral power, particularly in higher frequencies, as depicted in Figure 3A. Decreasing the cortical damping *γ* (as shown in Figure 3B) reduced the power of the EEG resonant frequency alpha while shifting its peak towards the lower frequencies. Furthermore, the corticothalamic delay *t*_*0*_ determined the locations of the resonant frequencies within the alpha and beta bands along the frequency axis, as observed in Figure 3C. These findings align with previous research (Rowe et al., 2004).

## 4 Discussion

This study explored the potential of training an MI classifier on a small set with augmented data, addressing the challenge of obtaining large datasets. Leveraging a computational model of the cortico-thalamic system (Robinson et al., 2002; Robinson et al., 2005; Kerr et al., 2008; Abeysuriya et al., 2015), we augmented EEG time series of MI epochs, leading to enhancements in classification accuracy across different DA strategies and augmentation factors. However, while the proposed DA method notably enhanced TP feature classification accuracy, it did not demonstrate similar improvements for the HFD feature. This observation suggests that the fitted CTM represents one feature more effectively than the other.

We believe that the model encountered challenges in accurately representing time-series-based features, particularly HFD. The CTM fitting process is based on spectra, adjusting model parameters until the analytical power spectrum matches the experimental one, as depicted in Figure 7. According to the Parseval theorem, the total energy remains identical whether calculated in the time or frequency domain. Consequently, the TP can be considered a spectra-based feature, as presented in Eq. 16. Since the fitting process is based on spectra, the TP of the simulated signals closely resembles that of the experimental ones. However, HFD relies on both phase and amplitude information from signal samples in the time domain. Unfortunately, during the fitting process, all phase information is lost, leading to a significant discrepancy between the HFD calculated from the simulated signals and the experimental ones.

Another notable aspect is the correlation between the success of NFT-based DA and the proficiency in MI. Specifically, when analyzing TP features among subjects with high MI proficiency (*Ac* ≥ 0.67), many DA strategies presented a significant accuracy improvement compared to the baseline. This observation suggests that our DA technique might have limited applicability among subjects with lower MI proficiency. It is common practice to exclude such subjects, as MI is a challenging task, and approximately 30% of the population fails to perform it completely (Alkoby et al., 2018). In these cases, for instance, the expected CSPs fail to manifest over the motor cortex (Ahn et al., 2018). Consequently, if a CSP time series poorly represents an MI condition, the augmented CSP time series would likely exhibit the same inadequacy and fail to contribute effectively to the classification process.

An alternative explanation could be attributed to the effect of diversity introduced by DA. When similar CSP time series generate comparable features with low discriminative power between MI conditions, it leads to poor classification accuracy. During the augmentation phase, diversification occurs in the time series produced by the fitted CTM. This variation among the generated time series contributes to enhanced classification. Therefore, if the initial time series are already quite similar, the introduced diversity in the generated time series of different conditions may lead to overlapping feature values, potentially worsening the classification. In essence, higher baseline accuracy implies more divergence in the time series and reduced overlap in the augmented features, thereby facilitating more substantial diversity contributions.

Several other insights can be drawn from the data presented in Table 1 and Table 2. Firstly, none of the DA strategies managed to attain the full set accuracy, potentially due to the strictness of the inverse CV approach employed. Alternatively, the ambitious reduction of the training set by a factor of three might have resulted in the insufficient representation of critical discriminative CSP characteristics within the baseline set, which were averaged out. This supposition is further supported by the observation that higher augmentation factors correlated with decreased accuracy improvement. This trend could indicate overfitting, a potential consequence of magnifying average discriminative traits, while overlooking more intricate ones. Furthermore, it is evident that parameter jittering adversely affected the classification accuracy of the augmented set. While only jittering *t*_*0*_ showed an accuracy rise above the baseline, it remained lower compared to the accuracy achieved without any jittering. This discrepancy might be attributed to the TP feature’s sensitivity to substantial changes in the power spectrum caused by alterations in *t*_*0*_, *γ*, or *α*, as previously discussed.

### 4.1 Comparison with other augmentation techniques

For both regular and professional subjects, the accuracy improvement from the noise-based DA method did not reach statistical significance. This technique operates under the assumption that experimental features adhere to a normal distribution, generating new features with increased variance. However, this assumption may not consistently hold true. Furthermore, the heightened variance introduced by this method could potentially result in feature values outside of realistic ranges. Each of these limitations might account for the ineffectiveness of this noise-based DA approach.

We also conducted a comparison with state-of-the-art MI DA techniques, as summarized in Table 3. Although our method showed a lower improvement in classification accuracy compared to other DA techniques, making a reliable comparison is challenging due to differences in MI classification frameworks and DA evaluation setups. For instance, while we employed a simple linear classifier to classify a single feature, many other studies utilized convolutional neural networks (CNNs), which typically yield higher baseline accuracy and are more responsive to DA. Additionally, our testing procedure involved inverse CV, which is more stringent compared to regular CV or a single validation set used in other studies. Furthermore, we augmented a small dataset, constituting 33% of the full one, whereas other studies often augmented the full dataset or a dataset reduced to only 50% of the full one in the best-case scenario. Despite most of the other studies using the ‘2a’ dataset for performance evaluation, these variations in methodologies make direct comparisons difficult.

**TABLE 3:** Comparison of our method to various state-of-the-art motor imagery DA techniques.

Researcher	Augmentation method	Dataset	Classification	Evaluation	Accuracy improvement (%)
Zhang et al. (2018)	Noise introduction in the frequency domain	“2a”	CNN [EEG time series]	20% validation	2.3
Gubert et al. (2020)	Extension of common spectral spatial patterns	“2a”	Fisher-LDA [CSP-based features]	50% validation	5.1
Lee et al. (2021)	Ensemble empirical mode decomposition	“2a”	CNN [Filter Bank CSP]	20% CV	8.2
Fahimi et al. (2021)	Deep convolutional generative adversarial network	“4a” (BCI comp. III)	deep-CNN [EEG time series]	50% validation	3.5
Our work	NFT	“2a”	LDA [CSP TP feature]	67% CV	2.1

It is worth noting that none of the mentioned DA techniques incorporate a physiological model. In fact, the advantages of our unique DA strategy are evident in the results. First, jittering was not essential to introduce variability in the generated time series. Despite fitting the model to an average spectrum across multiple epochs, the generated epochs displayed enough variability to diversify the training set. This capability arises from the CTM’s design, tailored to simulate EEG signals embedded with physiologically grounded noise. Secondly, when we did employ jittering, its extent was directly controlled using the model parameter *t*_*0*_. Altering axon-propagation delay causes a shift in resonance peaks along the frequency axis, consequently, impacting TP. Thus, employing the CTM allows for informed adjustment of augmented data by tuning parameters with a physiological meaning. Lastly, features extracted from NFT-augmented time series exhibit a distribution similar to experimental features, unlike those augmented with noise. This distinction arises from CTM’s ability to generate time series embodying typical EEG characteristics, encompassing features with realistic ranges and distributions.

### 4.2 Future directions

Moving forward, there are several avenues warranting further exploration in the realm of NFT-based DA. To begin with, while our investigation focused on TP and HFD features, the landscape of MI classification boasts a multitude of other features like kurtosis, sample entropy, and wavelet coefficients (Schiratti et al., 2018). It is plausible that the CTM’s ability to represent each feature differs, and not all features may be suitable for this augmentation technique. Further research in this direction could elucidate how the model encapsulates the distinct characteristics of each feature. Additionally, it would be intriguing to explore the application of this DA approach in an MI classification pipeline utilizing a deep-learning classifier that directly classifies time series rather than features. On one hand, DA might prove to be more efficient in this scenario, as deep-learning classifiers are more prone to overfitting, a problem that DA methods typically address. On the other hand, deciphering which features of the data are represented in the neural network poses a challenge, making it difficult to systematically analyze the DA process within the NFT model and its outcomes.

Secondly, a deeper investigation into parameter jittering is essential. Understanding the impact of jittering across different NFT parameters and different features, to discern why certain parameter jittering contributes to improved DA, while others do not, is critical. Moreover, delineating the optimal method for jittering each parameter—adjusting the range and distribution of jittered values–demands exploration.

Thirdly, there is potential in considering inter-epoch diversity and intra-epoch non-stationarity. Instead of fitting a CTM to the average of all subject epochs (per CSP, per MI condition), segmenting epochs based on certain criteria and fitting the CTM to subgroup averages might magnify the contribution of DA. Dividing the MI period of the epoch into smaller segments and fitting the CTM to each segment separately could be beneficial. For example, in the ‘2a’ dataset, EEG signal characteristics may change considerably during the MI period (from *t = 2.5* to *t = 5*); thus, segmenting this period could enhance augmentation performance by capturing these alterations. Moreover, we can also fit and augment event-related potentials that occur at the onset of motor imagery.

Finally, the scalability and adaptability of the augmentation approach should be tested across a larger subject pool. Exploring its adaptation for other BCI paradigms could also offer valuable insights into its broader applicability.

The DA method we have developed is poised for integration into various MI-based BCI systems to enhance classification accuracy and shorten MI training sessions. In fact, we have already successfully integrated it into the SES-BCI framework. Gathering feedback from other developers regarding the integration process, practical application, and the effectiveness of our DA method would be invaluable for further refinement and wider application.

## 5 Summary and conclusion

In this study, we introduced a novel DA method leveraging NFT for MI-based BCIs. Our approach utilized a CTM (Robinson et al., 2002; Robinson et al., 2005; Kerr et al., 2008; Abeysuriya et al., 2015) fitted to MI EEG epochs to generate artificial EEG epochs that amplified training-set diversity, thereby aiding improved MI classification. Our aim was to address the challenge of limited training data availability for MI classification. Along with this research, we developed the SES-BCI, designed to assist individuals with limited mobility in exploring their surroundings. Beyond its role as an integration platform for our DA method, this system was a continuous source of motivation throughout our study.

Several substantial findings emerged from this investigation. While the DA method significantly improved the accuracy of TP feature classification, it did not yield similar enhancements for HFD feature classification. This discrepancy suggests that the model is more adept at representing one feature over the other, potentially due to the nature of the fitted CTM. Moreover, the study observed a relation between MI proficiency and the efficacy of DA. Subjects with higher baseline accuracy tended to benefit more from the augmentation process, emphasizing the influence of initial proficiency on DA success.

While this study’s focus was on TP and HFD features, exploration of other MI-related features would offer a broader understanding of how the CTM represents each feature. Investigating different NFT parameters for jittering and accounting for inter-epoch diversity could enhance the method’s efficacy further.

In conclusion, this study demonstrates the promise of employing physiologically-inspired computational models to augment EEG time series in BCI paradigms. It underscores the need for a nuanced understanding of model-feature relationships and the influence of MI proficiency on augmentation effectiveness. This innovative DA approach offers significant potential for advancing MI-BCI systems, paving the way for continued research and development within the field, ultimately enhancing the quality of life for individuals with motor disabilities.

## Data Availability

Publicly available datasets were analyzed in this study. This data can be found here: https://bbci.de/competition/iv/download/.

## Abbreviations

BCI, Brain–computer interface; CNN, Convolutional neural network; CSP, Common spatial pattern; CTM, Corticothalamic NFT model; CV, Cross-validation; DA, Data augmentation; EEG, Electroencephalography; HFD, Higuchi fractal dimension; MI, Motor imagery; NFT, Neural field theory; SES-BCI, Surrounding exploring BCI system; SSVEP, Steady-state visual evoked potentials; TP, Total power.
